# *OsBTBZ1* Regulates Nitrate Transport in *Arabidopsis thaliana* via *NRT2.1* Modulation Under Salt Stress

**DOI:** 10.3390/ijms27188180

**Published:** 2026-09-14

**Authors:** Laurent Soulard, Nopphakhun Khunpolwattana, Chutarat Punchkhon, Triono Bagus Saputro, Luca Comai, Teerapong Buaboocha, Supachitra Chadchawan

**Affiliations:** 1 Center of Excellence in Environment and Plant Physiology, Department of Botany, Faculty of Science, Chulalongkorn University, Bangkok 10330, Thailand; laurent.soolard@gmail.com (L.S.); nopphakhun.kh@gmail.com (N.K.); lcomai@ucdavis.edu (L.C.); 2 Department of Biology, Faculty of Science, Chiang Mai University, Chiang Mai 50200, Thailand; chutarat.p@cmu.ac.th; 3 Biology Department, Faculty of Science and Data Analitics, Institut Teknologi Sepuluh Nopember, Campus ITS Sukolilo Surabaya, Surabaya 60111, Indonesia; trionobsaputro@bio.its.ac.id; 4 Department of Plant Biology and Genome Center, University of California at Davis, Davis, CA 95616, USA; 5 Center of Excellence in Molecular Crop, Department of Biochemistry, Faculty of Science, Chulalongkorn University, Bangkok 10330, Thailand; teerapong.b@chula.ac.th

**Keywords:** *OsBTBZ1*, nitrate limitation, NaCl stress, combining stress, *NRT2.1*

## Abstract

Nitrogen availability and salinity are two major environmental factors affecting plant growth and development. Nitrate uptake is strictly regulated through transcriptional networks that balance nutrient assimilation and stress responses. The role of BTB-ZF transcription factors, which have been implicated in various abiotic stress responses, in coordinating nitrate uptake remains largely unknown; specifically, the precise molecular mechanisms through which *OsBTBZ1*, a BTB-ZF transcription factor from *Oryza sativa*, confers salt tolerance remain to be fully elucidated. Here, we characterize the function of *OsBTBZ1* in *Arabidopsis thaliana* under nitrate and salt stress conditions. Transcriptomic analysis of the *bt3* mutant and revertant lines expressing *OsBTBZ1* in the *bt3* mutant background indicated a relationship with *NRT2.1*, which encodes a high-affinity nitrate transporter. The *bt3* mutant exhibited severe root growth inhibition under low nitrate and salt stress; however, expression of *OsBTBZ1* restored root growth under combined stress, supporting a role for *AtBT3* in stress adaptation. Our findings suggest that *OsBTBZ1* may contribute to the coordination of nitrate-responsive pathways and root plasticity under stress, potentially through regulation of nitrate transport-related processes, including those associated with *NRT2.1*. These findings highlight the potential of *OsBTBZ1*-mediated regulatory mechanisms for improving nitrogen use efficiency and stress tolerance in future crops.

## 1. Introduction

Nitrogen is a key macronutrient required for plant growth, and its availability influences physiological processes, including root architecture and stress responses [1]. In plants, nitrate is absorbed primarily through nitrate transporters such as *NRT2.1*, a high-affinity nitrate transporter involved in nitrogen uptake under limiting conditions [2,3]. The expression of *NRT2.1* is strictly regulated by transcription factors, ensuring optimal nitrate assimilation while balancing stress responses [4].

Salt stress disrupts nitrogen metabolism by impairing nitrate uptake and triggering oxidative stress [1]. Plants cope with salinity by activating stress-responsive transcription factors, which regulate both nutrient uptake and reactive oxygen species (ROS) detoxification pathways [5]. BTB-ZF transcription factors have been implicated in various abiotic stress responses, yet their role in coordinating nitrate uptake remains largely unknown. *OsBTBZ1*, a rice BTB-ZF transcription factor, shares sequence homology with Arabidopsis *AtBT3* and demonstrates the conserved function of BTB-ZF proteins in stress adaptation [6,7].

Recent studies have highlighted *OsBTBZ1* as a key regulator of salt tolerance in *Oryza sativa*. Chutimanukul et al. [8] conducted a comparative transcriptome analysis between the salt-sensitive cultivar KDML105 and salt-tolerant chromosome segment substitution line CSSL16, identifying *OsBTBZ1* among nine candidate genes associated with salt stress responses. *OsBTBZ1* has been proposed to function as a transcription factor, regulating downstream targets, which have not been clearly characterized. This was further supported by subcellular localization studies by Saputro et al. [7], which confirmed the nuclear enrichment of *OsBTBZ1*, aligning with its predicted transcriptional activity. Despite these insights, the precise molecular mechanisms through which *OsBTBZ1* confers salt tolerance remain to be fully elucidated.

In this study, we aimed to determine *OsBTBZ1* function using a heterologous system, *Arabidopsis thaliana*. We analyzed the transcriptomes of wild type (WT), the bt3 mutant line, the overexpression line, and the revertant line, which showed enrichment of nitrate transport-related genes. Therefore, we hypothesized that *OsBTBZ1* is involved in nitrate transport, especially under salt stress. Using transgenic lines with ectopic *OsBTBZ1* expression, we investigated its role in nitrate signaling and stress adaptation. Our findings reveal a novel dual function of *OsBTBZ1* in nitrate adjustment under salt stress conditions. We also discuss the importance of nitrate transport during salt stress in rice.

## 2. Results

### 2.1. K-Means Clustering of the Transcriptome Reveals the Gene Clusters Involved in Various Responses Under Salt Stress

**Figure 1:**
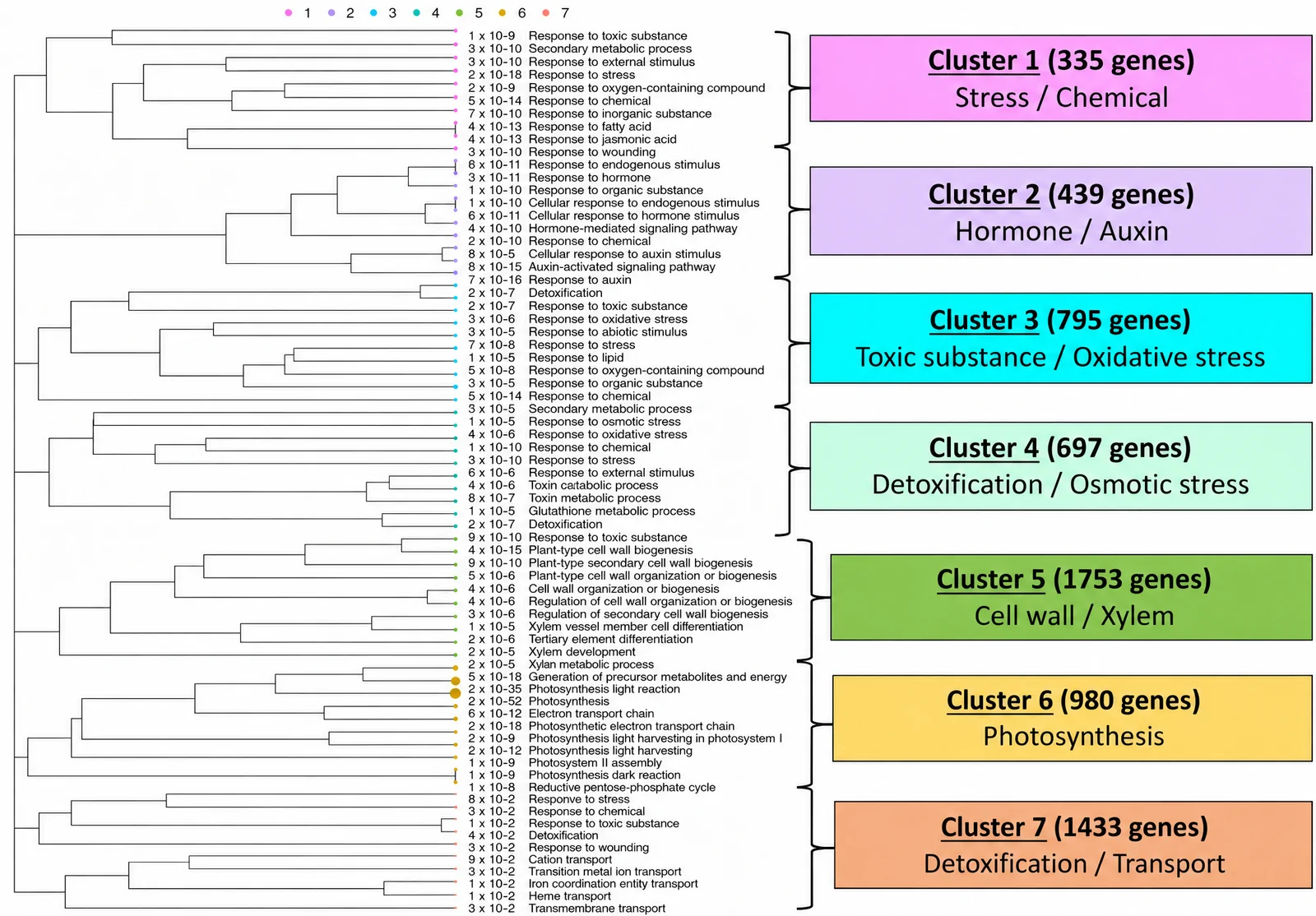
Phylogenetic distribution of “Biological Process” pathways across seven k-means clusters. The clusters were identified by k-means clustering of all DEGs identified across all comparisons (i.e., 6432 genes). For each cluster, the total number of genes involved and the top 10 pathways are displayed. For each pathway, the false discovery rate (FDR) value is shown.

A k-means clustering analysis was performed on all DEGs (Supplementary Table S1) identified across all comparisons (i.e., 6432 genes), and an enrichment analysis was performed to identify the most represented “Biological Process” pathways within each k-means cluster (Figure 1). Seven k-means clusters were identified. Gene cluster 1 was globally associated with functions related to the response to “stress” and “chemical”. Gene cluster 2 was globally associated with functions related to the response to “hormone,” particularly “auxin”. Gene cluster 3 was globally associated with functions related to the response to “toxic substance” and “oxidative stress”. Gene cluster 4 was globally associated with functions related to “detoxification” and “osmotic stress”. Gene cluster 5 was globally associated with functions related to “cell wall biogenesis” and “xylem differentiation”. Gene cluster 6 was globally associated with functions related to “photosynthesis”. Finally, gene cluster 7 was globally associated with functions related to “detoxification” and “transmembrane transport”.

### 2.2. Co-Expression Network Analysis Identified Key Gene Modules Regulated by OsBTBZ1

**Figure 2:**
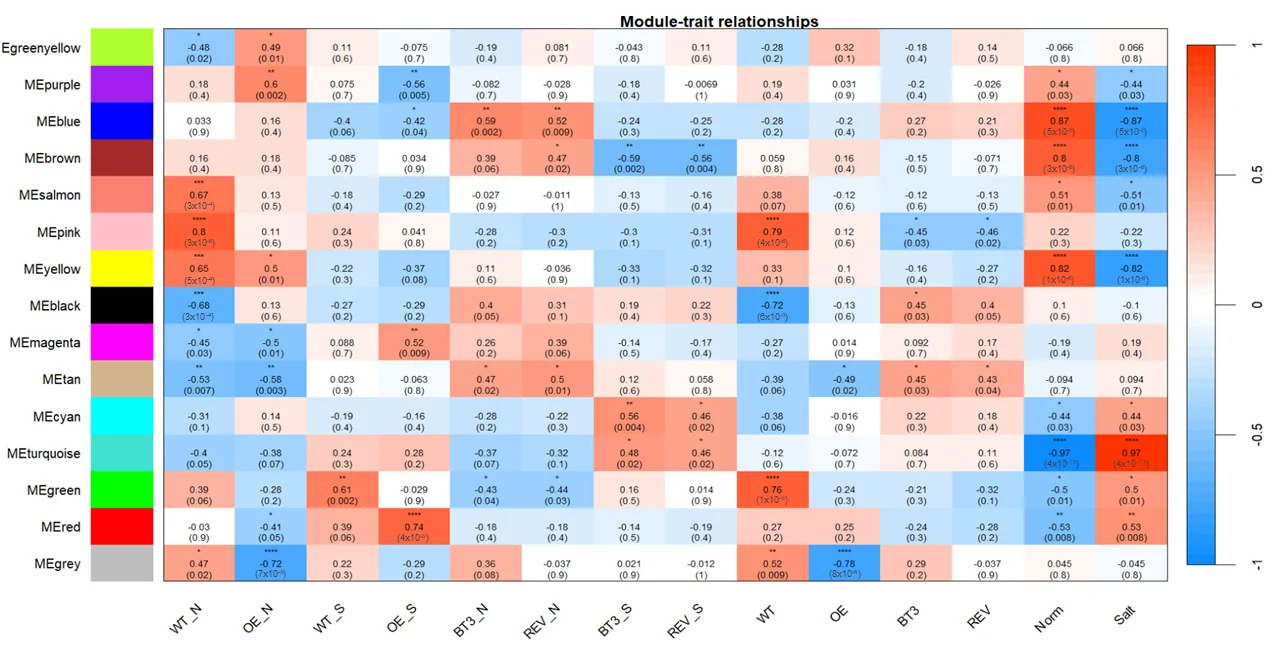
Correlation matrix of 15 WGCNA modules with experimental factors (genotype (WT: wild type, OE: overexpression of OsBTBZ1 line, BT3: bt3 mutant line, REV: revertant line of bt3 mutant with OsBTBZ1 expression), treatment (N: normal and S: salt stress), and genotype × treatment interactions). Red and blue indicate positive and negative correlations, respectively, with correlation coefficients and significance values displayed for each relationship. * represents *p* < 0.05, ** represents *p* < 0.01, *** represent *p* < 0.001,and **** represents *p* < 0.0001.

To elucidate the functional role of *OsBTBZ1* in nitrate signaling and salt stress adaptation, we conducted a comparative analysis of *A. thaliana* genotypes, including wild-type (WT), *OsBTBZ1* overexpression lines (OE-1), *AtBT3* knockdown mutants (*bt3*), and complemented lines (REV-1). Plants were subjected to normal and salt stress conditions (150 mM NaCl), followed by transcriptome analysis to identify DEGs and enriched functional pathways regulated by *OsBTBZ1*. To uncover the regulatory landscape influenced by *OsBTBZ1*, WGCNA was performed on 6432 DEGs identified across multiple genotypic and stress conditions [9]. This analysis revealed 15 distinct co-expression modules, each representing clusters of genes with correlated expression patterns. Among them, the “Magenta” module emerged as a strong candidate, displaying significant differential expression between *OsBTBZ1* OE lines and WT plants under both normal and salt-stress conditions (Figure 2).

### 2.3. Module–Trait Associations and Functional Enrichment of the Magenta Module

**Figure 3:**
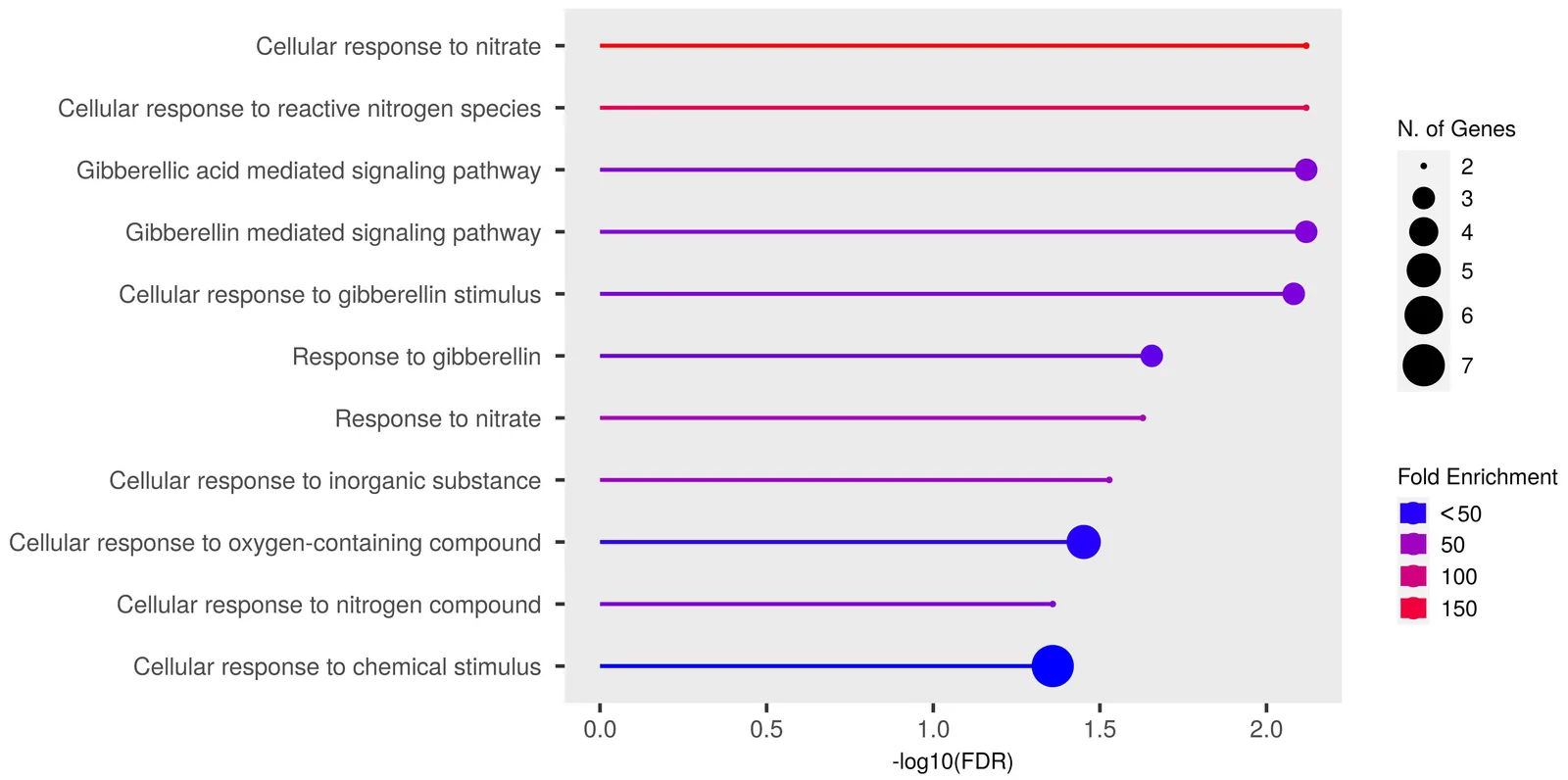
Functional enrichment of the “Magenta” WGCNA module. The top 20 enriched biological processes are ranked by false discovery rate (FDR) values. For each pathway, the number of genes and fold enrichment values are indicated. Pathways of particular interest are marked with a black star.

Correlation analysis between module eigengenes and experimental conditions highlighted key gene clusters responding to nitrogen and salt stress (Figure 3). Notably, there were 28 genes in the Magenta module, and this module showed strong positive correlations with OE_N and OE_S, while also distinguishing itself from WT and *bt3* mutant backgrounds. Functional enrichment analysis of this module (Figure 3, Table 1) identified pathways associated with nitrate signaling, reactive nitrogen species (RNS) detoxification, and gibberellin-mediated growth responses. Enrichment analysis revealed that *OsBTBZ1* influences genes involved in cellular responses to nitrate, gibberellin signaling, and RNS detoxification (Figure 3). Key genes in the Magenta module included *NIA1* and *NIA2*, which encode nitrate reductases essential for nitrogen assimilation, and *NRT2.1*, a high-affinity nitrate transporter crucial under nitrogen-limiting conditions. The strong correlation of these genes with *OsBTBZ1* expression suggests a role in regulating nitrate uptake and metabolic adaptation under stress. Additionally, *OsBTBZ1* appears to modulate gibberellin signaling, as indicated by the enrichment of ELF3, ELF4, and LHY, which integrate hormonal signaling with environmental responses.

### 2.4. AtBT3 Mutation Led to Main Root Growth Enhancement in Sufficient Nitrate (2 mM Nitrate), Whereas Deficient Nitrate Content Decreased Main Root Growth

Based on the WGCNA, the involvement of nitrate transport was suggested, leading to an investigation of the combined stress effects of salt and nitrate stress.

**Figure 4:**
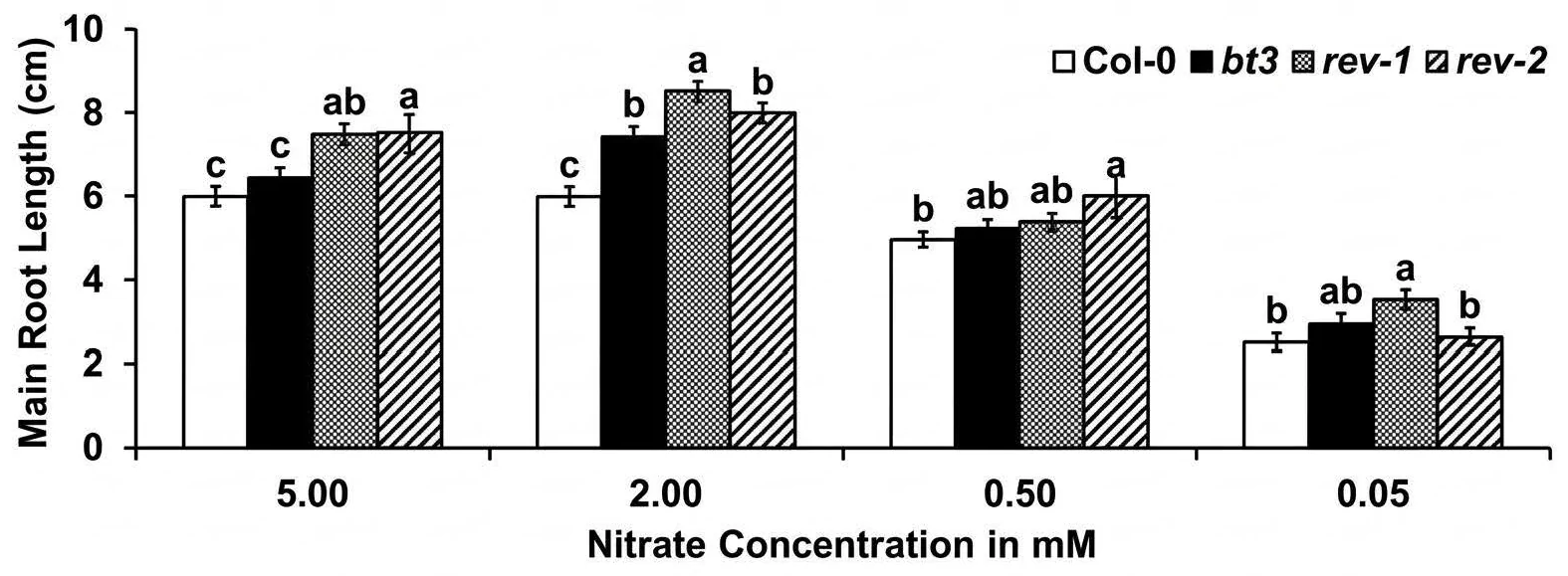
Root length response to varying nitrate conditions. Main root lengths (cm) of wild-type (Col-0), *AtBT3* mutant, and revertant lines (REV-1, REV-2) grown under different nitrate concentrations (control at 2.0 mM, high nitrate at 5 mM, and low nitrate at 0.5 mM). Bars represent means ± SE of at least six plants per genotype and condition. Different letters indicate significant differences (*p* < 0.05) within each condition, according to Duncan’s multiple range test.

Under sufficient nitrate conditions, the *bt3* mutant and ectopic expression lines with *OsBTBZ1* expression (REV-1 and REV-2) had significantly longer main roots compared with WT. The high-nitrate supplement (5 mM nitrate) did not enhance main root growth. Notably, REV-1 and REV-2 showed significantly less root length reduction compared with the WT and bt3-knockout mutant (Figure 4), indicating that the *OsBTBZ1* gene may confer partial resistance to the inhibitory effects of excess nitrate, potentially through indirect regulatory mechanisms or modulation of nitrate signaling pathways.

Under low-nitrate conditions (0.5 mM), all genotypes exhibited reduced root growth compared with normal nitrate levels. However, REV-1 and REV-2 lines maintained significantly longer roots than WT and *bt3* mutants (Figure 4), suggesting a beneficial role for *OsBTBZ1* in facilitating nitrate uptake or improving nutrient utilization efficiency under limited nitrate availability. Enhanced nitrate uptake efficiency is a critical trait for plant survival under nitrogen-limited conditions, with high-affinity nitrate transporters such as NRT2.1 playing essential roles [2,10,11].

**Figure 5:**
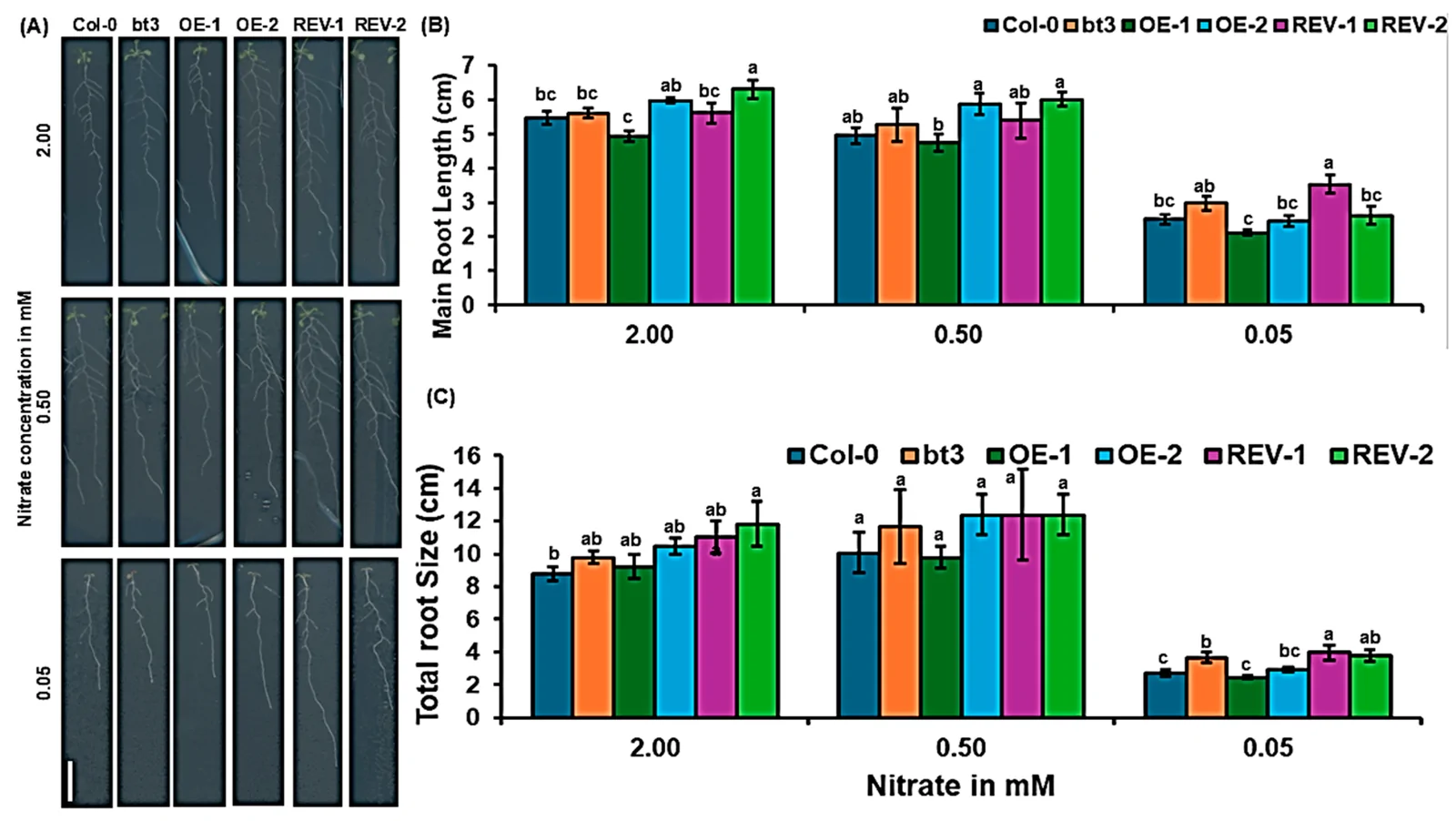
Root phenotypic responses of *Arabidopsis thaliana* Col-0 wild type (WT), *AtBT3* mutant (bt3), *OsBTBZ1* overexpression lines (OE-1, OE-2), and revertant lines (REV-1, REV-2) under varying nitrate conditions. (**A**) Representative images of root systems from WT, bt3, OE-1, OE-2, REV-1, and REV-2 grown on vertical agar plates containing control medium (2 mM nitrate), moderate nitrate insufficiency (0.5 mM), and severe nitrate insufficiency (0.05 mM). Images were captured 10 days post-germination. Scale bars: 1 cm. (**B**) Main root length measurements across different nitrate conditions. (**C**) Total root size across nitrate conditions. All data represent the mean ± SE of six biological replicates per genotype and condition. Different letters indicate statistically significant differences (*p* < 0.05) among genotypes within the same nitrate treatment, as determined by Duncan’s multiple range test.

Further experiments to investigate the lower nitrate level at 0.05 mM were performed (Figure 5). We determined both main root length and total root size of wild type (Col-0), bt3 mutant, OE1, OE2, REV1 and REV2, grown under normal nitrate level (2 mM), moderate nitrate insufficiency (0.5 mM) and severe nitrate insufficiency (0.05 mM) levels. Based on the overall phenotypes (Figure 5A), severe nitrate insufficiency strongly inhibited root growth, both main root length and lateral roots. Similar trends in responses to normal and moderate nitrate levels were observed in the bt3 mutant and revertant lines. The overexpression lines, OE1 and OE2, showed responses similar to WT and the bt3 mutant across all nitrate levels (Figure 5B,C). Only the revertant lines, REV1 and REV2, showed higher total root size than WT (Figure 5C).

### 2.5. Salt Stress Inhibited Main Root Length Growth

**Figure 6:**
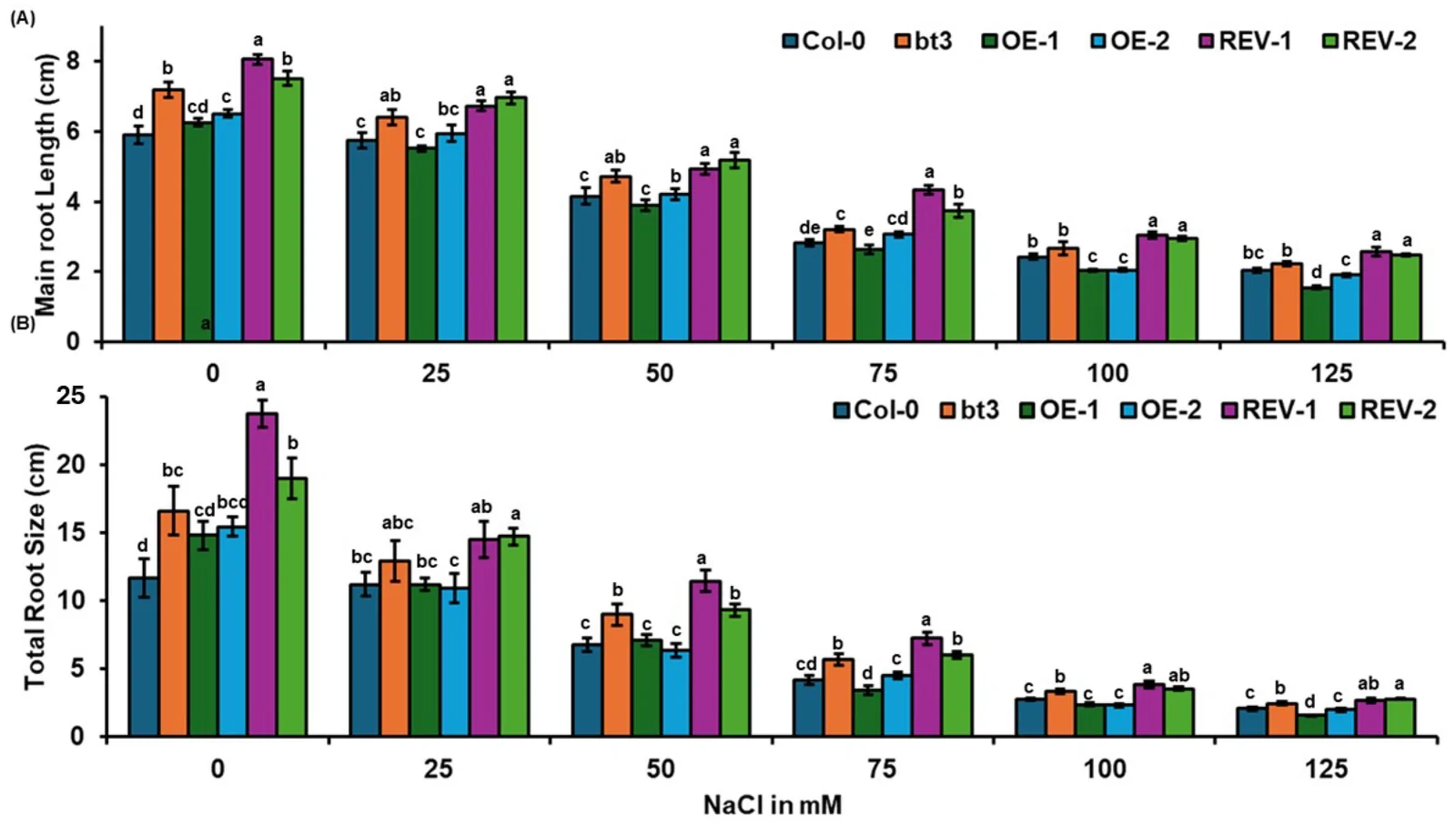
Main root length (cm) (**A**) and total root size (cm) (**B**) of WT, bt3, OE-1, OE-2, REV-1, and REV-2 grown under different salt stress conditions from 0 mM NaCl to 125 mM NaCl. Bars represent means ± SE of 6 plants per genotype and condition. Different letters indicate significant differences (*p* < 0.05) among genotypes within each treatment, as determined by Duncan’s multiple range test.

Supplementing the nutrient medium with increasing concentrations of NaCl (0, 25, 50, 75, 100 and 125 mM) generally inhibited root length across all genotypes, indicating a universal response to salinity-induced stress, rather than genotype-specific effects. Root length reduction due to NaCl supplementation was significant when compared with the control without NaCl supplementation (Figure 6A), suggesting a direct inhibitory effect of salt stress. This observation is consistent with known effects of salt stress, which induce osmotic and ionic stress and negatively affect cell elongation and division [12]. The inability of revertant lines (REV-1 and REV-2) to fully reverse root length reduction under these saline conditions suggests that OsBTBZ1 does not directly mitigate NaCl toxicity but instead indirectly influences nitrate-mediated root growth, possibly via nutrient signaling networks. However, both overexpression lines, OE1 and OE2, enhanced root length under no NaCl stress, but showed greater root growth inhibition at higher NaCl concentrations. The total root size amplified the salt stress responses in these lines (Figure 6A).

### 2.6. Ectopic Expression of OsBTBZ1 Could Reverse the Main Root Growth of bt3 Mutants Under Salt Stress Under Nitrate-Deficient Conditions

**Figure 7:**
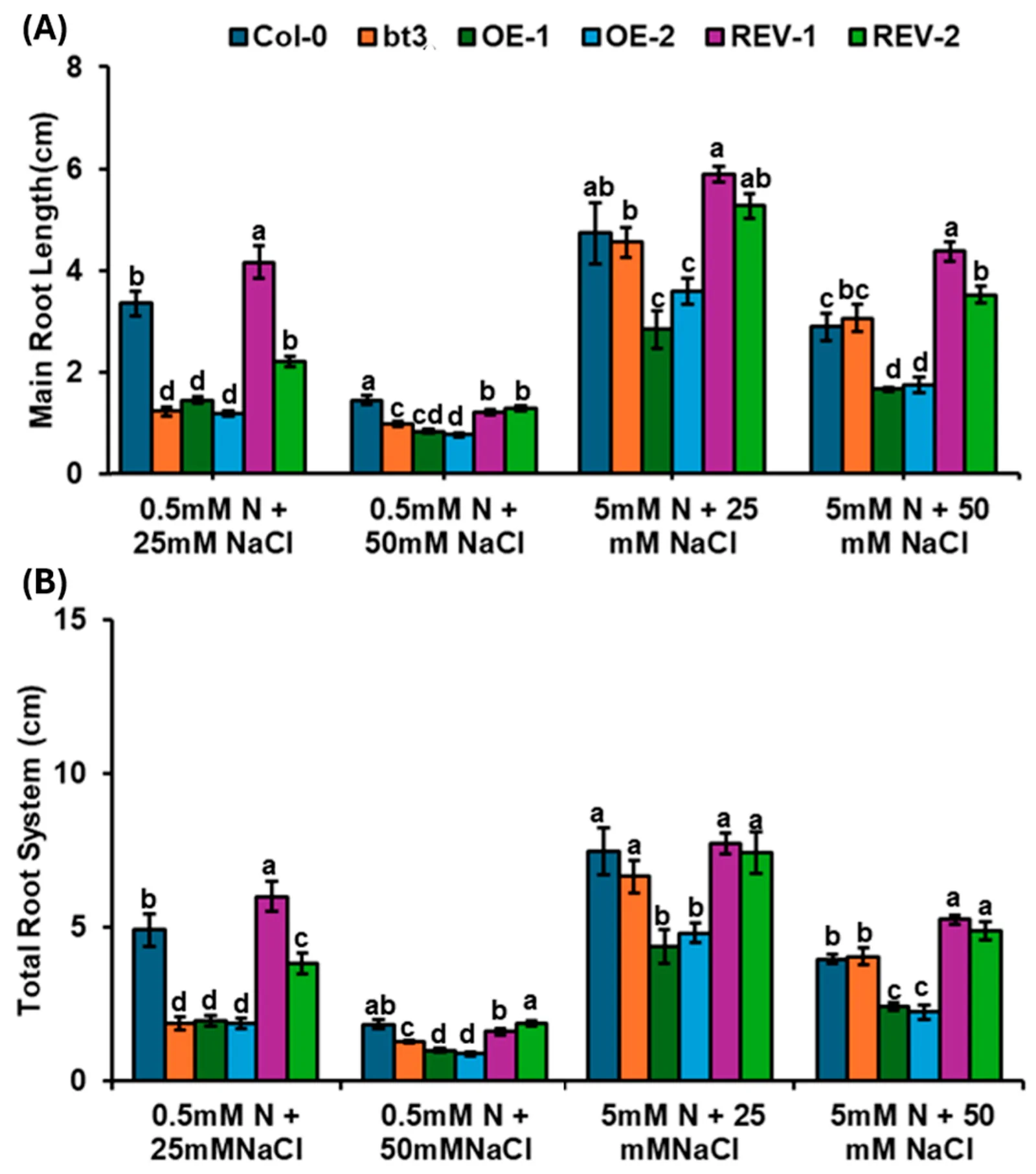
Main root length (**A**) and total root size (**B**) under combined moderately low nitrate (0.5 mM N) or sufficient nitrate (5 mM N) together with slight NaCl stress (25 mM NaCl) or NaCl stress (50 mM NaCl). Bars represent means ± SE (n = 6). Different letters denote significant differences (*p* < 0.05) according to Duncan’s multiple range test.

To investigate the combined effects of salt stress under nitrate-insufficient conditions, we selected two NaCl concentrations (25 mM and 50 mM) and two nitrate levels: moderate nitrate insufficiency (0.5 mM N) and high nitrate supplementation (5 mM N). Ten days after germination, we measured main root length and total root size. Both parameters were similar (Figure 7). At low NaCl stress (25 mM NaCl) and limited nitrate supplementation, root growth of the bt3 mutant and overexpression lines, OE1 and OE2, was strongly inhibited when compared to WT. On the other hand, the revertant lines, REV1 and REV2, could revert their growth to WT or higher than WT’s root growth (Figure 7A,B), but at a higher level of 50 mM NaCl, REV1’s and REV2’s main root length was higher than the bt3 mutant, but lower than WT (Figure 7A). For total root size, the revertant phenotypes were similar to WT (Figure 7B).

When the salt-stressed plants were grown under sufficient nitrate (5 mM N) conditions, the revertant lines’ main root length and total root size were similar to WT’s under 25 mM NaCl stress and significantly higher than WT’s under 50 mM NaCl (Figure 7). These results suggest that *OsBTBZ1* contributes positively to nitrate uptake or signaling under stress, possibly by enhancing expression or activation of nitrate transporters (e.g., *NRT2.1*). Furthermore, structural analyses indicate that *OsBTBZ1*’s extended N-terminal and internal insertion motifs might facilitate interactions with regulatory proteins involved in nitrate sensing and uptake, corroborating transcriptomic evidence of increased NRT2.1 expression.

### 2.7. Sequence Alignment and Conserved Domains Between OsBTBZ1 and AtBT3

**Figure 8:**
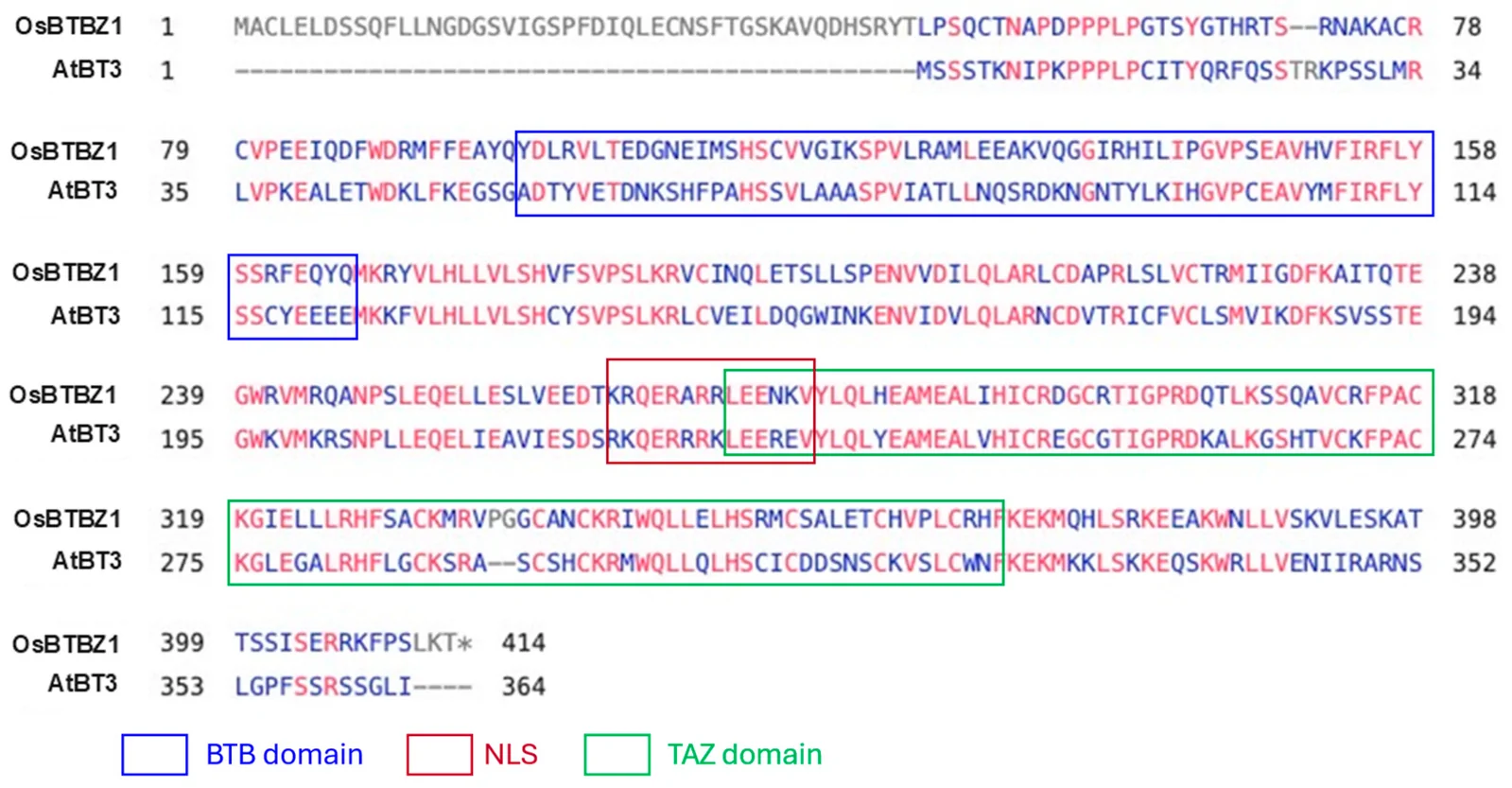
Pairwise sequence alignment between *OsBTBZ1* and *AtBT3* transcription factors reveals conserved functional domains. The amino acid sequence of *OsBTBZ1* (*O. sativa*, LOC_Os01g66890) was aligned with *AtBT3* (*A. thaliana*, AT1G05690) to assess sequence conservation. Identical residues are highlighted in red, indicating conserved regions critical for protein function. Gaps (-) represent insertions or deletions in sequence alignment. The BTB and TAZ domains, including the nuclear localization signal (NLS), are indicated by colored boxes.

We performed a global pairwise alignment between the amino acid sequences of *OsBTBZ1* (*O. sativa*, LOC_Os01g66890; NCBI RefSeq XP_015640130.1) and *AtBT3* (*A. thaliana*, AT1G05690; NCBI RefSeq NP_172060.2) [13]. The full-length *OsBTBZ1* protein comprises 413 amino acids, whereas *AtBT3* consists of 364 residues. The alignment revealed approximately 47% sequence identity and 60–65% similarity, including conservative substitutions, highlighting substantial evolutionary conservation (Figure 8). Length differences primarily resulted from an extended N-terminal region and minor internal insertions in *OsBTBZ1*. Despite sequence variation, both proteins clearly conserved critical functional domains. Both *OsBTBZ1* and *AtBT3* contain a conserved BTB/POZ domain (~120 amino acids), which mediates protein–protein interactions, notably with Cullin-based E3 ubiquitin ligases involved in ubiquitin-mediated proteolysis [6,14]. Both sequences feature a TAZ domain characterized by cysteine- and histidine-rich zinc-binding motifs. This domain facilitates interactions with transcriptional regulators and DNA, implicating *OsBTBZ1* and *AtBT3* in transcriptional regulation mechanisms [14]. This key interaction motif, featuring a conserved tryptophan residue flanked by basic amino acids, is preserved in *OsBTBZ1*, suggesting a similar regulatory mechanism. Beyond major domains, several shorter conserved motifs, including stretches enriched in basic residues (such as “RKQER…LEEREV” in *AtBT3* and “KRQER…LEENKV” in *OsBTBZ1*), were identified. These motifs potentially function as nuclear localization signals, essential for the nuclear function of these transcriptional regulators.

## 3. Discussion

### 3.1. OsBTBZ1 Acts as a Hub for Nitrate-Salt Stress Crosstalk

The WGCNA findings establish *OsBTBZ1* as a previously uncharacterized central regulator coordinating nitrate assimilation, reactive oxygen and nitrogen species (ROS/RNS) homeostasis, and hormonal signaling under stress conditions. Under non-stressed conditions, *OsBTBZ1* enhances nitrate assimilation by modulating nitrate reductases (*NIA1*/*NIA2*) while synchronizing nitrogen metabolism with circadian regulators such as *ELF3*, *ELF4*, and *LHY*. Previous studies have shown distinct yet complementary roles for *NIA1* and *NIA2* in Arabidopsis. Although *NIA1* expression is enriched predominantly in leaf tissues and spongy mesophyll cells, *NIA2* is strongly expressed in meristematic regions such as shoot apical meristems and cambial cells, indicating a critical role in nitrate assimilation associated with active growth [15]. Furthermore, *nia1/nia2* double mutants exhibit severely reduced biomass production and compromised root elongation compared with WT plants under nitrate-limiting conditions, highlighting the essential role of these genes in plant adaptation to nitrogen deficiency [15]. AtSIBP1, a BTB domain-containing protein, has been reported to play a role in salt stress signaling [16]. Under salt stress, we report for the first time that *OsBTBZ1* directly or indirectly upregulates the high-affinity nitrate transporter NRT2.1 to sustain nitrate uptake, thereby mitigating stress-induced growth inhibition. Previous studies have highlighted the importance of NRT2.1 in high-affinity nitrate transport under low-nitrogen conditions [2,10,11]. In apple, the 14-3-3 protein MdGRF11 interacts with the BTB protein MdBT2 to modulate anthocyanin accumulation under nitrate deficiency [17]. Collectively, these findings identify *OsBTBZ1* as a novel transcriptional regulator integrating nitrogen metabolism and hormonal pathways to maintain root growth and facilitate plant adaptation to concurrent nitrate deprivation and salt stress. As nitrate and salt stress are tightly interconnected, *OsBTBZ1* may serve as a crucial regulator of nitrogen homeostasis, optimizing nitrate uptake to sustain growth under high salinity.

### 3.2. OsBTBZ1 Functionally Complements AtBT3 in Enhancing Salt Tolerance

The superior performance of REV-1 and REV-2 compared with all other genotypes suggests that *OsBTBZ1* effectively compensates for the loss of *AtBT3* function under salt stress. The full complementation of *OsBTBZ1* in REV-1 and REV2 lines suggests that *OsBTBZ1* activity is optimized in the *bt3* mutant background. This suggests that *OsBTBZ1* and *AtBT3* are functionally redundant. However, the overexpression lines inhibited root growth under combined salt and nitrate insufficiency, suggesting that proper *BTBZ1* expression levels are important for regulating other genes. The divergent effects of *OsBTBZ1* in *Arabidopsis* WT vs. *bt3* mutant exemplify how a single BTB-ZF factor can tip this balance. Properly modulated, *BT3/OsBTBZ1* constrains nitrate uptake to optimize root growth under stress or low-N conditions. Our findings align with emerging models in which nitrate signaling and salt-stress homeostasis are interconnected. Given this, *AtBT3* and *OsBTBZ1* likely coordinate these crosstalk pathways. By integrating low nitrate and salt stress, BTB-ZF proteins ensure that root elongation is sustained only when the nutritional environment is favorable—a delicate equilibrium that can be perturbed by misexpression but restored by complementation of the defective BT3 network.

Rice is considered the ammonium-preferred crop, especially under flooded conditions [18]. However, salt stress usually occurs in drying soil, where evaporation pulls the salt solution in the deeper soil upward. This creates aerobic conditions in the topsoil; nitrification converts NH_4_^+^ to NO_3_^−^, increasing nitrate availability [18]. As a result, nitrate transporters like NRT2.1 have become more important.

### 3.3. Functional Conservation and Divergence of OsBTBZ1 and AtBT3

The BTB domain is a highly conserved structural motif identified across diverse taxa, including plants, animals, and certain fungi [19]. Structurally, this domain typically consists of approximately 116 amino acid residues arranged into five α-helices, three β-sheets, and characteristic N- and C-terminal extension regions [20,21]. The BTB domain mediates protein–protein interactions predominantly through homo- and heterodimerization, enabling diverse regulatory roles within cellular signaling networks [22]. Such structural diversity underscores the versatility and complexity of BTB proteins in regulating multiple biological processes and stress response pathways. BTB and TAZ domain scaffold proteins play a crucial role in Arabidopsis development [23]. The high conservation of key residues within BTB and TAZ strongly suggests functional similarity between OsBTBZ1 and AtBT3, supporting the hypothesis that OsBTBZ1 could complement or enhance AtBT3 functions in Arabidopsis. Specifically, conserved residues within the BTB domain suggest similar involvement in ubiquitin-mediated proteolysis pathways, which are crucial for regulating protein stability under stress conditions [6,14]. Stogios et al. [19] described the BTB domain as a critical protein–protein interaction module that participates in diverse cellular processes, including transcriptional control, cytoskeletal organization, ion channel modulation, and mediating ubiquitination-dependent protein degradation.

The preservation of the TAZ zinc finger domain further implies conserved transcriptional regulatory roles, possibly through DNA binding or interaction with other transcription factors. These findings suggest that OsBTBZ1 likely exerts its regulatory roles through both transcriptional control and ubiquitin-mediated protein turnover.

## 4. Materials and Methods

### 4.1. Plant Materials

Four independent Arabidopsis lines were selected for analysis: wild-type Columbia-0 (Col-0, WT), two independent *OsBTBZ1* overexpression lines (OE_1), two independent *OsBTBZ1* complementation lines in the *bt3* background (REV-1), and the *bt3* knock-down mutant. All transgenic lines used in this experiment were generated as described in Saputro et al. [7]. All transgenic lines (T_3_ generation) were verified for homozygosity through segregation analysis on hygromycin-containing selective medium, confirming the presence of a single transgene insertion.

### 4.2. Transcriptomic Analysis

Transcriptomic profiling was conducted to investigate the regulatory roles of *OsBTBZ1* and *AtBT3* under normal and salt stress conditions. Four *Arabidopsis thaliana* genotypes were selected for this study: wild-type (Col-0), OsBTBZ1-overexpressing lines in the wild-type background (OE-1), the *AtBT3* knock-down mutant line (*bt3*), and revertant lines expressing *OsBTBZ1* against the *bt3* mutant background (REV-1). The seeds were sterilized and vernalized for 2 days, then grown on square Petri dish plates with half-strength Murashige and Skoog (MS) medium for 7 days and then transferred and grown on vertical plates with full-strength MS medium (normal conditions) or MS medium supplemented with 150 mM NaCl (salt-stress conditions) [24]. Whole 12-day-old seedlings (three independent biological replicates, each replicate containing pooled seedlings per genotype and treatment) were harvested and immediately preserved in RNAlater^®^ stabilization solution (Invitrogen, Carlsbad, CA, USA) at room temperature (25 °C) to prevent RNA degradation. In total, 24 RNA samples (4 genotypes × 2 treatments × 3 biological replicates) were submitted to the University of California, Davis, USA, for sequencing on the Illumina NovaSeq platform. Differentially expressed genes (DEGs) were identified using DESeq2 software (v.1.24.0) [25]. Genes exhibiting significant differential expression (adjusted *p* < 0.01) between genotypes and treatments were considered for further analysis. Quality assessments of sequencing data were conducted using principal component analysis (PCA) and data distribution plots to confirm sample consistency and reproducibility. The transcriptome dataset was submitted to NCBI with accession no. PRJNA1506070.

### 4.3. Weighted Gene Co-Expression Network Analysis (WGCNA)

To identify clusters of co-expressed genes linked to genotype and stress conditions, a weighted gene co-expression network analysis (WGCNA) was performed using the R platform [9]. Prior to analysis, read counts were normalized and variance-stabilizing-transformed (VST) using DESeq2 to ensure data quality and reduce technical noise, as confirmed by PCA. The transformed dataset was subsequently analyzed using WGCNA with a soft-thresholding power of 7 and a minimum module size of 20 genes. This approach yielded 15 distinct color-coded modules, each representing highly correlated gene groups exhibiting genotype-specific and stress-responsive expression patterns.

### 4.4. Gene Ontology (GO) Enrichment Analysis

Functional enrichment analysis of identified WGCNA modules was performed using ShinyGO (version 0.77) software, focusing on GO categories related to “Biological Process.” The top 20 significantly enriched GO terms for each module were identified based on false discovery rate (FDR)-adjusted *p*-values to highlight the biological processes and pathways most relevant to *OsBTBZ1* and *AtBT3*-mediated nitrate–salt stress responses.

### 4.5. Seed Sterilization, Sterile Plate Culture, and Growth Conditions

Arabidopsis seeds were surface-sterilized by immersing them in absolute ethanol for 1 min, followed by treatment with a sterilization solution containing 2.8% (*w*/*v*) sodium hypochlorite and 0.1% (*v*/*v*) and 0.02% (*v*/*v*) Tween-20 for 5 min under sterile conditions. The seeds were then rinsed four to five times with sterile distilled water and stratified in the dark at 4 °C for 48 h to synchronize germination. Stratified seeds were sown aseptically onto 12 × 12-cm square Petri dishes containing a defined nutrient medium [26]. The growth medium consisted of 0.5% (*w*/*v*) sucrose, 1% (*w*/*v*) agar, and 0.2 mM MES/Tris (pH 5.6). The nutrient compositions for all experimental conditions are detailed in the Supplementary Tables. The control medium contained minimally sufficient macronutrient levels. For nitrate-limitation treatments, the nitrogen concentration was reduced from 2 mM to 0.5 and 0.05 mM, with Na^+^ and Cl^−^ concentrations adjusted accordingly to maintain ion balance. Additional treatment conditions are provided in Table S1. All nutrient solutions were prepared from stock solutions using ddH_2_O. Micronutrients (from a 1000× concentrated stock solution) (Table S2) were supplied at the following final concentrations: 0.025 μM CuSO_4_, 0.068 μM ZnSO_4_, 0.2 μM MnSO_4_, 0.453 μM H_3_BO_3_, 0.13 μM (NH_4_)_6_Mo_7_O_24_, and 0.12 μM CoCl_2_ [25]. Growth media were autoclaved at 121 °C for 20 min, and 50 mL of medium was dispensed into each square Petri dish. Plants were grown vertically in a controlled-environment growth chamber under a 9-h photoperiod, with a light intensity of 120 μE m^−2^ s^−1^, 60% relative humidity, and a constant temperature of 20 °C.

### 4.6. Root Phenotyping

Plates were scanned from the back (through the agar) at 600 dpi using a flatbed Epson scanner (Epson, Tokyo, Japan) to capture high-resolution root images at 6–10 days after germination (DAG). Images were saved in JPEG format for subsequent analysis. Root traits were quantified using EZ-Rhizo (version 2.4.8.5) [27,28]. Individual root structures were detected and quantified using the software’s detection tool. All extracted data were recorded along with metadata, including plant age, growth medium composition, genotype, and treatment conditions. Root growth parameters were analyzed in Microsoft Excel, then compiled in Root-Vis 2 [28] for further statistical analysis.

### 4.7. Data Analysis and Statistics

Root system architecture (RSA) data collected from scanned images were initially organized, and data processing was performed in Microsoft Excel. Subsequent statistical analyses were performed using IBM SPSS Statistics (version 28.0, IBM Corp., Armonk, NY, USA). Differences between experimental groups were assessed using Duncan’s multiple range test (DMRT). Statistical significance was determined at *p* < 0.05, and significant differences among treatments or genotypes are clearly indicated in the respective figures.

## 5. Conclusions

This study revealed *OsBTBZ1* as a critical transcriptional regulator that coordinates nitrate signaling and root growth adaptations under nitrogen limitation and salt stress. Through comprehensive transcriptomic and phenotypic analyses, we demonstrate that *OsBTBZ1* functionally complements Arabidopsis *AtBT3*, highlighting an evolutionarily conserved regulatory role for BTB-ZF transcription factors in stress resilience. Specifically, *OsBTBZ1* enhances nitrate uptake efficiency by positively regulating the expression of the high-affinity nitrate transporter NRT2.1, thereby facilitating sustained root growth under salt stress. Elucidating these molecular mechanisms would establish a detailed framework for understanding *OsBTBZ1*’s broader regulatory network. Collectively, our findings position *OsBTBZ1* as a promising candidate gene for developing crops with enhanced salt tolerance and improved nitrogen use efficiency. Harnessing this transcriptional regulator through genetic engineering and molecular breeding approaches could significantly contribute to sustainable agriculture and food security under increasingly challenging environmental conditions.

## Figures and Tables

**Table 1:** Differentially expressed genes (DEGs) in the WGCNA “Magenta” module under stress conditions. Genes listed belong to the “Magenta” co-expression module identified by WGCNA and met the criteria for differential expression under the conditions tested.

Pathway	Genes
GO:0071249 cellular response to nitrate	NRT2:1 ANR1
GO:1902170 cellular responses to reactive nitrogen species	NRT2:1 ANR1
GO: 0010167 response to nitrate	NRT2:1 ANR1
GO: 0071241 cellular response to inorganic substance	NRT2:1 ANR1
GO: 0009740 gibberellic acid mediated signaling pathway	GASA5 AT4G30410 PIF3
GO: 1901699 cellular responses to nitrogen compound	NRT2:1 ANR1
GO: 0010476 gibberellin-mediated signaling pathway	GASA5 AT4G30410 PIF3
GO: 0071370 cellular response to gibberellin stimulus	GASA5 AT4G30410 PIF3
GO: 0009739 response to gibberellin	GASA5 AT4G30410 PIF3
GO: 1901701 cellular responses to oxygen-containing compound	NRT2:1 GASA5 AT4G30410 ANR1 PIF3
GO: 0070887 cellular response to chemical stimulus	NRT2:1 PMSR2 PIN5 GASA5 AT4G30410 ANR1 PIF33

## Data Availability

Our data are available at PRJNA1506070 in NCBI.

## Supplementary Materials

The following supporting information can be downloaded at https://www.mdpi.com/article/10.3390/ijms27188180/s1.

## Abbreviations

The following abbreviations are used in this manuscript:

DAG	days after germination
DEGs	differentially expressed genes
DMRT	Duncan’s multiple range test
FDR	false discovery rate
GO	gene ontology
MS	Murashige and Skoog
NLS	nuclear localization signal
OE	overexpression
PCA	principal component analysis
RNS	reactive nitrogen species
ROS	reactive oxygen species
VST	variance-stabilizing-transformed
WGCNA	weighted gene co-expression network analysis
WT	wild-type
