# Supplementary material for: OsBTBZ1 Regulates Nitrate Transport in Arabidopsis thaliana via NRT2.1 Modulation Under Salt Stress
Source: Int J Mol Sci. 2026 Sep 14;27(18):8180. doi: 10.3390/ijms27188180 (PMC13607357; doi:10.3390/ijms27188180)
Supplement: Supplementary file 1 [file ijms-27-08180-s001.zip › Supplementary data_BTBZ1 Project.pdf]

Supplementary data

Table S2. Composition of Plant Macronutrients. Each medium was prepared with 1 mL of 1,000× micronutrient stock solution, supplemented with 0.5% (w/v) sucrose and 1.0% (w/v) agar. The pH was adjusted to 5.6 using 0.2 M MES/Tris buffer. (Kellermeier *et al*., 2014).

Reagent	Stock in M	Normal (Control) (ml/L)	Nitrogen deficiency (0.5 mM) (ml/L)	Nitrogen deficiency (0.05 mM) (ml/L)	Salt stress (25 mM) (ml/L)	Salt stress (50 mM) (ml/L)	Nitrogen deficiency (0.5 mM + 25 mM NaCl) (ml/L)
$CaCl_2$	0.50	0.5	0.5	0.5	0.5	0.5	0.5
$MgSO_4$	0.25	1	1	1	1	1	1
KNO_3	1.00	2	0.5	0.05	2	2	0.5
NaH_2PO_4	0.50	1	1	1	1	1	1
Fe(III)Na_2-EDTA	0.0425	1	1	1	1	1	1
$MgCl_2$	0.1	2.5	2.5	2.5	2.5	2.5	2.5
NaCl	1.00	0	0	0	25	50	25
KCl	1.00	0	1.5	1.95	0	0	1.5

Table S3. Micronutrient Stock Solution Composition. The micronutrient stock solution (1,000×) was prepared according to standard plant tissue culture formulations. Each component was dissolved in distilled water and stored at 4 °C in amber bottles to prevent light-induced degradation. For all culture media described in Table 1, 1 mL of this micronutrient stock solution was added per liter of basal medium**,** yielding the final concentrations shown in the table.

Reagent	Amount (g/L)	Final Concentration (in 1× medium)
Manganese(II) sulfate monohydrate ($MnSO_4 \cdot H_2O$)	0.3042	2.5 μM Mn^{2+}
Boric acid (H_3BO_3)	2.78	25 μM B
Zinc sulfate heptahydrate ($ZnSO_4 \cdot 7H_2O$)	0.1093	2 μM Zn^{2+}
Ammonium molybdate tetrahydrate ($(NH_4)_6Mo_7O_{24} \cdot 4H_2O$)	0.0185	0.05 μM Mo
Copper(II) sulfate monohydrate ($CuSO_4 \cdot H_2O$)	0.0399	0.5 μM Cu^{2+}
Cobalt(II) chloride hexahydrate ($CoCl_2 \cdot 6H_2O$)	0.0024	0.02 μM Co^{2+}
